# NPF-Driven *Gart* Expression Fuels Gut Absorption and Modulates Feeding via a Negative Feedback Loop

**DOI:** 10.3390/insects17050528

**Published:** 2026-05-21

**Authors:** Lei He, Qin Wei, Yifei Guo, Qingqing Li, Zhangwu Zhao

**Affiliations:** 1 Shanxi Key Lab Nucl Acid Biopesticides, Institute of Applied Biology, Shanxi University, Taiyuan 030006, China; 18307050605@163.com (Q.W.); 19735027809@163.com (Y.G.); 13073566637@163.com (Q.L.); 2 School of Life Sciences, Shanxi University, Taiyuan 030006, China

**Keywords:** Neuropeptide F, GART trifunctional enzyme, feeding, food absorption

## Abstract

Energy homeostasis requires precise coordination between brain-derived appetitive signals and peripheral nutrient-handling mechanisms. Although Neuropeptide F (NPF) and its mammalian homolog NPY are well-established central stimulators of feeding, whether and how they regulate nutrient assimilation in the gut remains unknown. Here, using *Drosophila*, we identify a previously unrecognized transcriptional circuit between NPF and the purine synthesis enzyme GART trifunctional enzyme (*Gart*) that governs feeding by controlling gut absorptive efficiency. We show that NPF signaling acts via its receptor NPFR to positively regulate *Gart* expression specifically within the intestine. Conversely, *Gart* activity exerts negative feedback on NPF expression, forming a reciprocal regulatory loop. Functionally, gut-specific, but not glial or fat body-specific, *Gart* is necessary and sufficient for promoting food absorption and consumption. Genetic epistasis experiments demonstrate that *Gart* acts downstream of NPF to execute its function. Strikingly, peripheral NPF from the fat body and gut, rather than brain-derived NPF, serves as the primary systemic signal driving this loop. Our findings reveal a gut-centered homeostatic module where NPF activates *Gart* to boost nutrient absorption, while the resultant feeding activity in turn curbs the signal, ensuring calibrated energy intake. This work redefines a canonical neuropeptide’s role from a pure behavioral driver to a key regulator of peripheral metabolic efficiency, and establishes a novel framework for understanding gut–brain communication in energy balance.

## Simple Summary

To maintain normal survival, animals must balance the energy they take in from food with what they use. The brain sends hunger signals to encourage eating, but how those signals actually help the body absorb nutrients from food is not well understood. Using *Drosophila*, we discovered a new communication pathway between the brain and the gut. We found that a feeding-promoting signal called NPF works directly on the gut to turn on a key metabolic enzyme named GART. This enzyme helps the gut absorb nutrients more efficiently. Surprisingly, once absorption happens, the gut sends a signal back to reduce the original brain signal, creating a self-balancing loop. This prevents overeating and ensures energy is taken up correctly. Our findings show that hunger signals do more than just make us want to eat—they also fine-tune how well the digestive system works. Understanding this loop may help people develop better strategies for managing conditions linked to eating, such as obesity or malnutrition, in humans.

## 1. Introduction

The maintenance of energy homeostasis is a fundamental biological challenge, requiring organisms to precisely balance nutrient intake with expenditure. This balance is governed by a complex interplay between central neural circuits that drive motivated behaviors, such as foraging and feeding, and peripheral metabolic organs that execute nutrient acquisition, processing, and storage [1,2,3]. Although significant progress has been made in decoding the brain-derived signals that stimulate or terminate eating, the molecular mechanisms by which these signals orchestrate adaptive changes in peripheral nutrient assimilation remain less understood.

The neuropeptide system is a phylogenetically conserved master regulator of energy balance. In mammals, NPY is a potent orexigenic (appetite-stimulating) signal released from the hypothalamus during states of energy deficit [4,5,6]. Its functional homolog in *Drosophila*, Neuropeptide F (NPF), similarly promotes feeding behaviors, especially under conditions of hunger or stress [7,8,9,10,11]. NPF signaling plays an important role in coordinating feeding behavior with metabolic need. The canonical view posits that NPF peptides primarily act on central circuits to modulate motivational drive [12]. However, emerging evidence suggests these peptides also exert direct effects on peripheral tissues, influencing glucose and lipid metabolism [13,14,15]. Although the intestine is the ultimate site of nutrient uptake, the pathways through which systemic NPF signals, integrated to fine-tune its absorptive capacity in response to whole-body energy demands, are entirely unknown.

GART trifunctional enzyme (*Gart*) is a critical component of the de novo purine synthesis pathway [16,17,18,19]. Purines are essential not only as building blocks for DNA and RNA but also as central components of cellular energy currency (ATP) and signaling molecules [20,21,22]. As such, *Gart* activity is intrinsically linked to cellular proliferation, bioenergetic capacity, and overall metabolic flux. Beyond its well-established role in development, we have previously shown that *Gart* operates in multiple peripheral tissues, including glia, the fat body, and gut, to regulate feeding rhythm and to control the storage and mobilization of energy reserves (glycogen and triglycerides), thereby contributing to systemic energy homeostasis [23]. These findings position *Gart* as a peripheral metabolic integrator to coordinate feeding behavior with energy needs.

Both NPF and *Gart* are involved in the regulation of feeding and energy homeostasis. Here, using *Drosophila melanogaster* as a model, we detected and discovered a previously uncharacterized regulatory circuit that operates within the gut to control nutrient absorptive efficiency through the gut–brain axis regulated by the NPF–*Gart* pathway. Our findings shift the paradigm of how a conserved neuropeptide regulates feeding, from a purely central driver of motivation to a peripheral coordinator of metabolic efficiency, and reveal a novel homeostatic feedback loop that ensures calibrated energy intake.

## 2. Materials and Methods

### 2.1. Fly Strains and Rearing

Flies were raised at 25 °C and 65% humidity under a 12 h light:12 h dark cycle. Three-to-five-day-old flies were used for experiments.

The [*T2A*-Gal4]*Gart*/cyo and 10 × UAS-*Gart*-V5 was generated by CRIPSPR-Cas9 [23]; the *repo*-Gal4/TM3 (BDSC: 7415), *nsyb*-Gal4 (BDSC: 51635) and *nsyb*-Gal80 (BDSC: 92154) were purchased from the Bloomington Drosophila Stock Center (Bloomington, IN USA); the *CG*-Gal4 was obtained from Junzheng Zhang’s lab (Beijing, China); the UAS-*NPF*-RNAi (THU2569) and UAS-*NPFR*-RNAi (THU2116) were purchased from the TsingHua Fly Center (Beijing, China); the *MyoIA*-Gal4 was obtained from Yi Rao’s lab (Beijing, China); the UAS-*Gart*-RNAi (VDRC: 46293) was purchased from the VDRC stock center (Wien, Austria). All lines in this study were outcrossed with *w^1118^* for at least 6 generations. All experiments were conducted using male flies.

### 2.2. RNA Isolation, RT—PCR and qRT—PCR

The brain, fat body and gut of 50 flies or whole bodies of 30 flies were dissected on ice, and were collected by liquid nitrogen (Taineng Gas Co., Ltd., Taiyuan, China) for RNA preparation. The protocols for RNA extraction, cDNA synthesis and qRT-PCR are described by He et al. [23]. The results were conducted according to threshold cycle (Ct) value based on the 2^−ΔΔCT^ method [24]. The primers are shown in Table S1.

### 2.3. Feeding Assay

The feeding assay was modified from our previous publication [23]. Briefly, male flies aged 3–5 days were entrained at 25 °C in LD for 3 days. The flies were switched from normal food to blue-dye food (100 mL of normal food (0.8% agar, 3.32% yeast, 3.16% sucrose, 6.32% glucose, 7.77% corn meal, 0.5% propionic acid, and distilled water) containing 2.5 g of FD&C Blue No. 1 [McCormick] (ROHA Dyechem (SHANGHAI) Co., Ltd., Shanghai, China)) for 2 h at different time points, after which they were frozen in liquid nitrogen, and decapitated. The samples were homogenized in 1000 μL ddH_2_O (Beijing Solarbio Science & Technology Co., Ltd., Beijing, China) and centrifuged (13,000 rpm) for 15 min. The supernatant was passed through a 0.22 μm syringe filter (Tianjin Keyilong Lab Equipment Co., Ltd., Tianjin, China) to remove debris and lipids and transferred to a new tube. The absorbance at 625 nm was measured by SpectraMax i3x (Meigu Biotechnology (Zhejiang) Co., Ltd., Wenzhou, China). The feeding levels were also normalized to the absorbance value/25 flies. The value of OD625 represents the intake of food.

### 2.4. Food Absorption Rate

Newly eclosed flies were maintained in a 25 °C LD cycle for 3 days and then transferred to diet containing blue dye for 24 h. Twenty-five flies were immediately frozen in liquid nitrogen as the pre-feeding baseline (recorded as A1). Another 25 flies were transferred to transparent diet (Beijing Solarbio Science & Technology Co., Ltd., Beijing, China) (containing 1% agar and 5% sucrose), fed freely for 6 h, then they were frozen in liquid nitrogen, decapitated, and collected as the post-feeding sample (recorded as A2). All samples were homogenized in 1000 μL ddH_2_O and centrifuged (13,000 rpm) for 15 min. The supernatant was passed through a 0.22 μm syringe filter to remove debris and lipids and transferred to a new tube. Absorbance at 625 nm was measured using a SpectraMax i3x microplate reader. Food absorption was calculated as described previously [25].

Absorption rate = (A1 − A2)/A1 × 100%

### 2.5. Quantification and Statistical Analysis

Analyses were performed using Microsoft Excel or Prism 10.4.0 (GraphPad), and graphs were plotted using Prism 10.4.0. For comparison among different genotypes or treatment groups, pairwise analyses were conducted by unpaired t test for two groups and one-way ANOVA for multiple groups of data. Data are represented as the mean ± SEM unless otherwise noted. Significance is indicated in the figure legends.

## 3. Results

### 3.1. Gut-Specific Gart Positively Regulates Feeding Through Absorption Efficiency

**Figure 1:**
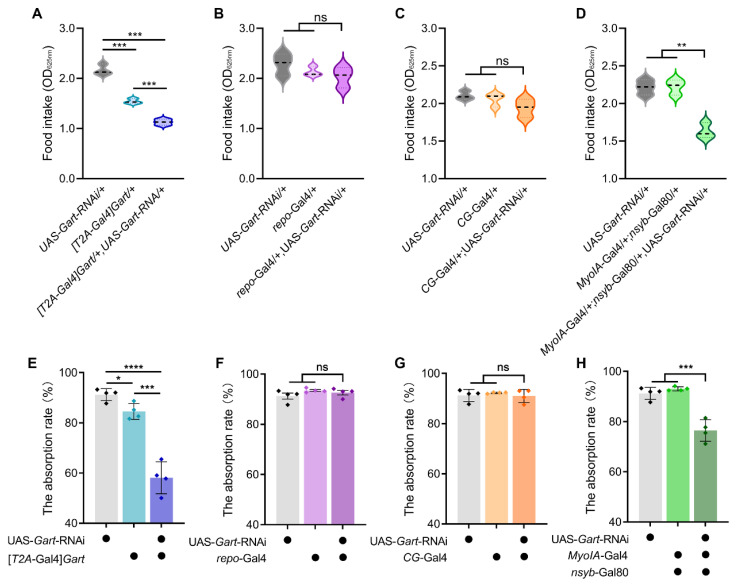
The effect of *Gart* deficiency on feeding behavior and food absorption rate of *Drosophila melanogaster*. (**A**–**D**) The food intake of flies with downregulated *Gart* in whole body (**A**), glial cells (**B**), fat body (**C**) and gut (**D**). (**E**–**H**) The food absorption rate of flies with downregulated *Gart* in whole body (**E**), glial cells (**F**), fat body (**G**) and gut (**H**). Black dots indicate the presence of this component in flies. The graphs show the mean ± SEM (*t* test; * *p* < 0.05, ** *p* < 0.01, *** *p* < 0.001, **** *p* < 0.0001; ns not significant). N = 3 biological replicates (25 ± 5 flies/repeat).

To determine whether and how *Gart* regulates feeding behavior, we first examined feeding of a heterozygous *Gart* mutant ([*T2A*-Gal4]*Gart*/+) (homozygous *Gart* knockout is lethal) and flies with further downregulation of *Gart* expression in heterozygous *Gart* mutant background ([*T2A*-Gal4]*Gart*/+; UAS-*Gart*-RNAi/+). Quantification of food intake revealed a significant decrease in feeding concomitant with reduced *Gart* expression (Figure 1A), indicating that *Gart* promotes feeding. Given the broad expression of *Gart*, we next sought to identify the tissue responsible for this metabolic function. Using tissue-specific RNAi drivers (*repo*-Gal4 for glia, *CG*-Gal4 for fat body, and *MyoIA*-Gal4; *nsyb*-Gal80 for the gut), we knocked down *Gart* in distinct tissues. Downregulation of *Gart* in either glia or the fat body had no discernible effect on feeding (Figure 1B,C). In striking contrast, gut-specific *Gart* knockdown recapitulated the low food intake phenotype of the *Gart* deficiency mutant (Figure 1D). These results indicate *Gart* predominantly functions in the gut.

We reasoned that intestinal *Gart* might modulate feeding by altering nutrient assimilation. To test this, we measured the food absorption rate. Consistent with the feeding phenotype, systemic reduction in *Gart* significantly impaired absorption efficiency (Figure 1E). Tissue-specific analysis demonstrated that this defect was exclusively attributable to the loss of *Gart* in the gut, while its knockdown in glia or the fat body was without any effects (Figure 1F–H). Collectively, these findings demonstrate that *Gart* promotes feeding primarily by enhancing nutrient absorption in the gut, positioning gut-specific *Gart* as a key determinant of feeding efficiency.

### 3.2. Peripheral NPF Is the Main Signal Source That Activates the Expression of Gart

**Figure 2:**
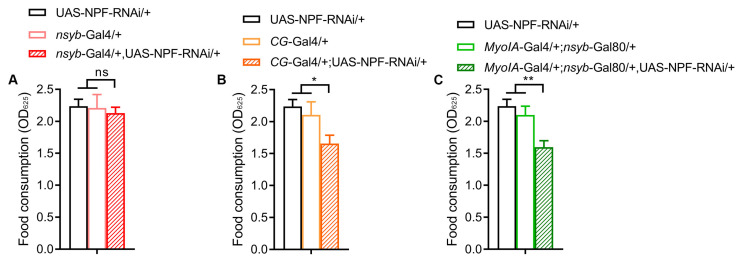
The effect of *npf* deficiency on feeding behavior of *Drosophila melanogaster*. (**A**–**C**) The food intake of flies with downregulated *npf* in brain (**A**), fat body (**B**) and gut (**C**). The graphs show the mean ± SEM (*t* test; * *p* < 0.05, ** *p* < 0.01; ns not significant). N = 3 biological replicates (25 ± 5 flies/repeat).

**Figure 3:**
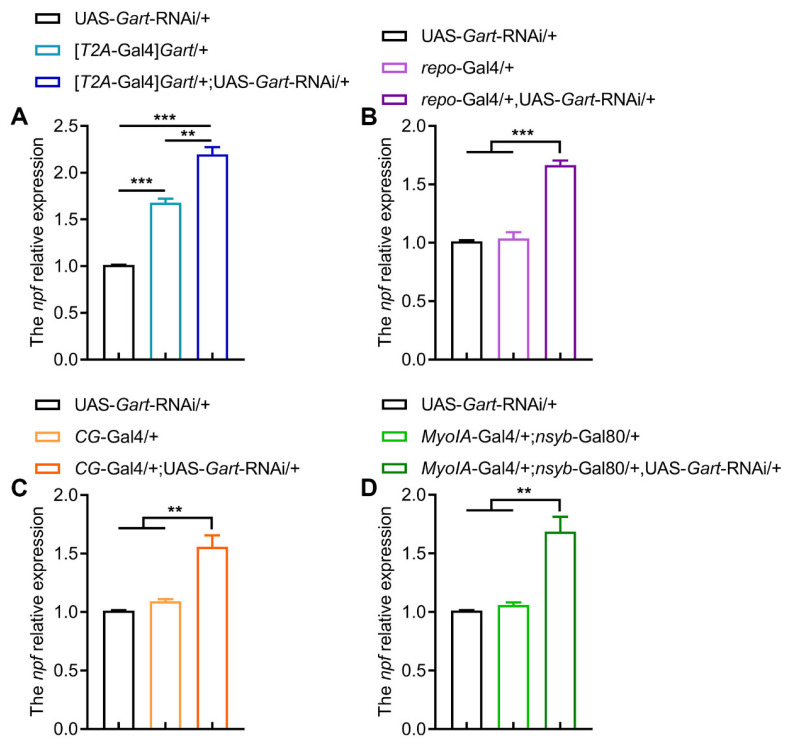
The effect of *Gart* deficiency on NPF expression levels. (**A**–**D**) The NPF expression levels of flies with downregulated *Gart* in whole body (**A**), glial cells (**B**), fat body (**C**) and gut (**D**). The graphs show the mean ± SEM (*t* test; ** *p* < 0.01, *** *p* < 0.001). N = 3 biological replicates (20 ± 5 flies/repeat).

To delineate the relationship between NPF and *Gart*, we first assessed their tissue-specific contributions to feeding. Strikingly, the brain-specific knockdown of NPF (*nsyb*-Gal4) had no effect on food intake, while knockdown of NPF in either the fat body (*CG*-Gal4) or the gut (*MyoIA*-Gal4; *nsyb*-Gal80) significantly reduced feeding (Figure 2A–C), indicating that peripheral (fat body and gut) NPF is a key driver of feeding behavior. Subsequently, we explored the molecular interplay between NPF and *Gart*. In flies with globally reduced *Gart* expression, we observed a corresponding upregulation of NPF transcript levels (Figure 3A). This negative feedback was recapitulated by tissue-specific *Gart* knockdown in glia, fat body and gut (Figure 3B–D), indicating that *Gart* represses NPF expression in multiple tissues.

**Figure 4:**
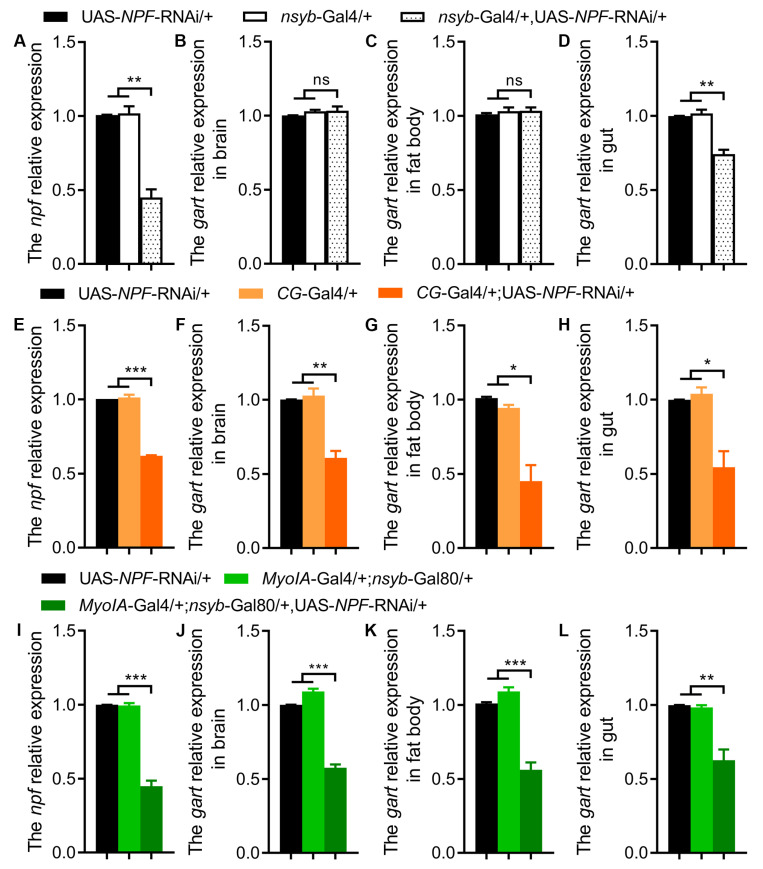
The effect of NPF deficiency on *Gart* expression levels. (**A**) The NPF expression levels of flies with downregulated *npf* in brain. (**B**–**D**) The *Gart* expression levels in brain (**B**), fat body (**C**) and gut (**D**) of flies lacking *npf*. (**E**) The NPF expression levels of flies with downregulated *npf* in fat body. (**F**–**H**) The *Gart* expression levels in brain (**F**), fat body (**G**) and gut (**H**) of flies lacking *npf*. (**I**) The NPF expression levels of flies with downregulated *npf* in gut. (**J**–**L**) The *Gart* expression levels in brain (**J**), fat body (**K**) and gut (**L**) of flies lacking *npf*. The graphs show the mean ± SEM (*t* test; * *p* < 0.05, ** *p* < 0.01, *** *p* < 0.001; ns not significant). N = 3 biological replicates (20 ± 5 flies/repeat).

**Figure 5:**
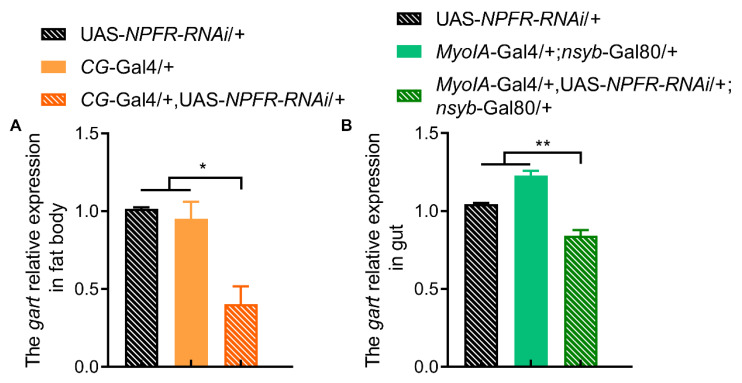
The effect of NPFR deficiency on *Gart* expression levels. (**A**,**B**) The *Gart* expression levels in fat body (**A**) and gut (**B**) of flies lacking *npfr*. The graphs show the mean ± SEM (*t* test; * *p* < 0.05, ** *p* < 0.01). N = 3 biological replicates (20 ± 5 flies/repeat).

Conversely, we investigated whether NPF regulates *Gart*. Brain-specific NPF knockdown selectively reduced gut-specific *Gart* expression without affecting its levels in the brain or fat body (Figure 4A–D). In contrast, NPF knockdown in the fat body or gut led to a pronounced downregulation of *Gart* not only in the targeted tissue but also systemically, including in the brain (Figure 4E–L). This demonstrates that peripheral NPF is the primary positive regulator of *Gart* expression across tissues. Furthermore, fat body- or gut-specific knockdown of the NPF receptor (NPFR) similarly reduced *Gart* levels (Figure 5A,B), confirming that NPF signals through NPFR to maintain *Gart* expression.

Collectively, these data reveal a core peripheral regulatory axis: NPF/NPFR signaling positively regulates *Gart* expression, while *Gart*, in turn, provides negative feedback on NPF. This reciprocal relationship is central to feeding control; furthermore, peripheral NPF serves as the dominant source for activating systemic *Gart* expression.

### 3.3. Gart Acts as the Essential Metabolic Effector of NPF in Feeding Regulation

**Figure 6:**
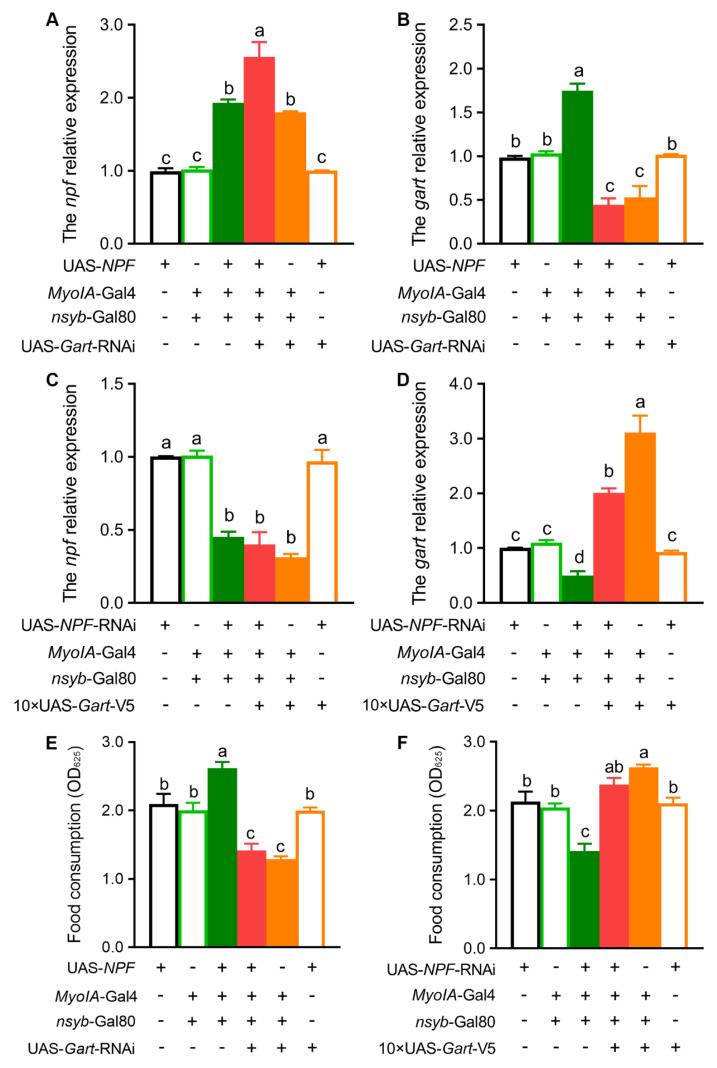
*Gart* rescues the impact of NPF deficiency on fruit fly feeding. (**A**,**B**) In the background of NPF overexpression, downregulation of *Gart* results in the expression levels of NPF (**A**) and *Gart* (**B**). (**C**,**D**) In the background of NPF downregulation, overexpression of *Gart* results in the expression levels of NPF (**C**) and *Gart* (**D**). (**E**) In the background of NPF overexpression, downregulation of *Gart* results in the food intake. (**F**) In the background of NPF downregulation, overexpression of *Gart* results in food intake. The graphs show the mean ± SEM (one-way ANOVA; the data with different lowercase letters in the column show significant differences). N = 3 biological replicates (25 ± 5 flies/repeat).

To definitively establish the genetic hierarchy between NPF and *Gart*, we performed a series of rescue experiments at the transcriptional and functional levels. First, we manipulated their expression in opposite directions. As expected, overexpression of NPF led to a significant increase in *Gart* transcript levels (Figure 6A,B). Crucially, when *Gart* was simultaneously knocked down in this NPF-overexpression background, the *Gart* mRNA level was reduced (Figure 6B), confirming that NPF acts upstream to promote *Gart* expression. Conversely, in a background of NPF knockdown, overexpression of *Gart* successfully elevated *Gart* mRNA without rescuing the low NPF levels (Figure 6C,D), consistent with *Gart* functioning downstream. Subsequently, we asked whether *Gart* is required for the physiological function of NPF. Overexpression of NPF robustly increased food intake, as anticipated (Figure 6E). Strikingly, this high feeding phenotype was completely abolished when *Gart* was knocked down concurrently, and feeding levels significantly decreased further (Figure 6E). Reciprocally, the decrease in feeding caused by NPF knockdown was fully reversed by co-overexpression of *Gart* (Figure 6F). These behavioral rescue experiments showed that *Gart* is a downstream effector of NPF. Collectively, these genetic and behavioral assays demonstrated that *Gart* is both sufficient and necessary to mediate the effects of NPF on feeding.

### 3.4. Gart Rescues the Impact of NPF Deficiency on Absorption Rate

**Figure 7:**
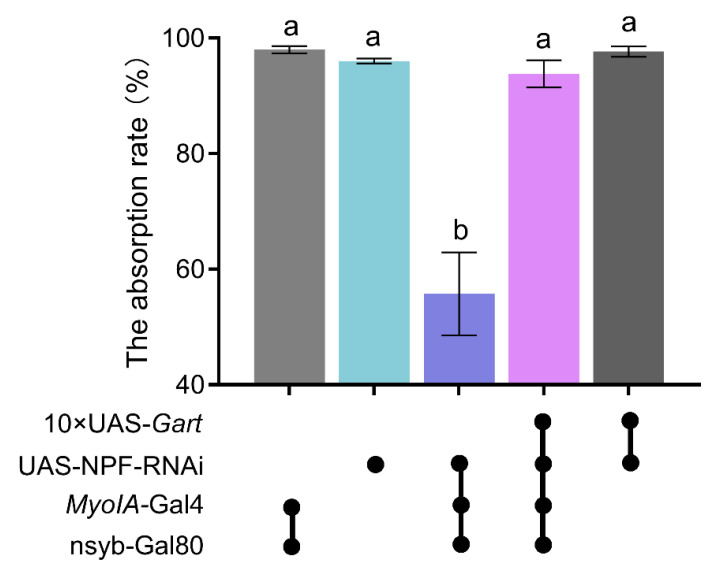
*Gart* rescues the impact of NPF deficiency on fly food absorption rate. The graphs show the mean ± SEM (one-way ANOVA; the data with different lowercase letters in the column show significant differences). N = 5 biological replicates (25 ± 5 flies/repeat).

To directly test whether the NPF-*Gart* axis converges on nutrient absorption, the mechanistic basis of feeding regulation, we measured the food absorption rate under genetic perturbations of this pathway. Consistent with the low feeding phenotype, knockdown of NPF significantly reduced the food absorption rate (Figure 7). Critically, this absorptive defect was fully rescued by concurrent overexpression of *Gart* (Figure 7). This rescue experiment provides definitive functional evidence that *Gart* acts downstream of NPF to modulate intestinal absorption efficiency. Thus, NPF promotes feeding primarily by upregulating gut-specific *Gart*, which in turn enhances nutrient assimilation.

Collectively, these findings establish a novel gut-centric homeostatic module wherein feeding is controlled through the precise regulation of nutrient absorption. We demonstrate that the purine synthesis enzyme, *Gart*, acts specifically within the intestine to enhance food absorption, thereby promoting feeding. This key metabolic effector is regulated by a reciprocal transcriptional loop with the neuropeptide NPF: peripheral NPF signaling, originating dominantly from the fat body and gut, acts through its receptor NPFR to upregulate *Gart* expression systemically, while *Gart* in turn provides negative feedback on NPF levels. Genetic epistasis analyses confirm that *Gart* is both necessary and sufficient to execute the effects of NPF on feeding and absorption, placing it as the essential downstream effector. Thus, our work redefines a conserved hunger signal from a pure behavioral driver to a coordinator of peripheral metabolic efficiency, revealing a dynamic feedback circuit that calibrates energy intake by modulating gut absorptive capacity.

## 4. Discussion

Feeding behavior represents the primary determinant of energy intake, serving as the fundamental behavioral interface between an organism and its environment [23,26,27]. Through foraging and consumption, animals secure the caloric resources necessary for survival, growth and reproduction [28]. However, the act of feeding alone does not guarantee energy acquisition; rather, it is nutrient absorption within the gut that constitutes the true gateway through which ingested food is converted into bioavailable energy [29]. The efficiency of this absorptive process ultimately dictates how much of the consumed nutrients are assimilated to meet systemic metabolic demands [30]. Despite its centrality, the molecular mechanisms by which absorptive efficiency is dynamically regulated to match whole-body energy needs have remained poorly understood. This study establishes a previously unrecognized homeostatic circuit that directly links a conserved orexigenic signal to the control of gut absorptive capacity. We demonstrate that NPF and *Gart* form a reciprocal transcriptional loop within peripheral tissues that governs feeding by tuning the efficiency of nutrient absorption in the gut. We therefore propose a dynamic working model. Under energy demand, peripheral NPF signaling is induced, which positively drives *Gart* expression in the gut via its receptor NPFR. Elevated *Gart* boosts purine metabolic flux, potentially enhancing enterocyte energetics (e.g., ATP levels) and/or nutrient transporter capacity, thereby increasing nutrient absorption. As absorption proceeds and energy status improves, accumulated *Gart* or its metabolites transcriptionally repress NPF, which reduces feeding by preventing over-activation of the absorptive program. Although our findings demonstrate that NPF in peripheral tissues (gut) modulates feeding behavior by affecting gut absorption capacity, NPF expressed in the central nervous system plays a more critical role in feeding regulation, possibly by integrating gustatory signals or coordinating energy homeostasis. Future studies using tissue-specific knockdown of NPF in the CNS will help dissect its distinct roles relative to peripheral NPF. This “feed-forward-driven, feedback-braked” loop enables an automatic transition from “accelerated absorption” to “homeostatic maintenance,” ensuring precise coupling between energy harvest and physiological need. This model carries significant physiological and evolutionary implications. It represents a strategy of “precision nutrition,” where organisms adapt to environmental flux not only by modulating “how much they want to eat” (appetite) but also by dynamically tuning “how much they can absorb” (efficiency). Such a reciprocal “neuropeptide–core metabolic enzyme” circuit may represent an evolutionarily conserved homeostatic principle. Analogous push–pull systems exist in mammals, where hunger signals like ghrelin and satiety signals like leptin/peptide YY (PYY) interact [31,32,33]. Our NPF-*Gart* module provides a simpler, potentially more ancient, cellular/organ-level analog for rapid metabolic tuning. Placing a fundamental housekeeping enzyme like *Gart* under dynamic transcriptional control by a neuropeptide offers a fresh perspective on metabolic adaptation. In addition, this work establishes gut absorptive efficiency as a critical and tunable regulatory dimension of energy homeostasis, and reveals how a neuropeptide signal can orchestrate metabolic output at the level of the absorbing organ itself.

Notably, our work fundamentally shifts the focus of feeding regulation from the central nervous system to the gut—the ultimate site of nutrient assimilation. Despite being broadly expressed across multiple tissues, *Gart* functions in a highly tissue-specific manner: only its loss in the gut, but not in glia or the fat body, leads to a concurrent reduction in both food intake and absorptive efficiency. This tissue-specific requirement suggests that gut-specific *Gart* governs feeding behavior by modulating nutrient absorption, thereby generating a peripheral “satiety” or “metabolic repletion” signal that is relayed to the brain to curtail further food consumption. These findings establish the gut as a key decision-making node in energy homeostasis and reveal that absorptive efficiency itself can serve as a feedback signal to regulate central feeding circuits. This mechanism operates in complementary parallel to central hunger/satiety circuits. In addition, a key finding is the dominant role of peripheral NPF in initiating this loop. NPF derived from the fat body and gut, rather than the brain, serves as the primary systemic signal to activate *Gart* expression across tissues, including the brain. This is consistent with research in *Ostrinia furnacalis*, where gut-derived NPF is the central regulator of feeding, operating through the activation of the PI3K signaling pathway [13]. Functionally, peripheral NPF serves as a systemic “metabolic accelerator” that drives nutrient absorption, whereas the *Gart*-mediated negative feedback acts as a “brake” to prevent excessive energy intake. This locally established gut-centric regulatory loop likely influences central feeding decisions through absorbed nutrients or gut-derived humoral signals, thereby constituting an integrated model of “peripheral metabolic circuit-to-central behavioral output” that orchestrates systemic energy homeostasis.

In summary, this study delineates a novel and physiologically coherent gut–brain circuit for feeding regulation, centered on a peripheral NPF–*Gart* reciprocal transcriptional loop that directly governs intestinal absorptive efficiency. This work fundamentally repositions the intestine as a primary executive target of the conserved orexigenic signal NPF, moving beyond its canonical central role. This research provides the first mechanistic evidence of a direct transcriptional feedback loop linking a neuropeptide to a core metabolic enzyme, thereby forging a definitive molecular bridge between behavioral drive and cellular energetics. Furthermore, this finding conceptually establishes “nutrient absorptive efficiency” as a critical, tunable dimension of feeding control, revealing a refined physiological strategy for adaptive energy harvest. Collectively, these findings offer a new molecular framework and a dynamic homeostatic model for understanding how inter-organ communication precisely calibrates energy balance. However, several important questions remain. First, what is the precise mechanistic link between *Gart* and absorption? How does peripheral NPF signaling communicate with the brain to downregulate central *Gart*? Is it via a humoral factor or a vagal-like neural pathway? Addressing these questions will not only refine this axis but may also offer novel insights into metabolic diseases, such as whether dysregulation of the human NPF/NPY system similarly disrupts gut function to contribute to obesity or malnutrition.

## Data Availability

The original contributions presented in this study are included in the article/Supplementary Materials. Further inquiries can be directed to the corresponding authors.

## Supplementary Materials

The following supporting information can be downloaded at: https://www.mdpi.com/article/10.3390/insects17050528/s1, Table S1: Primer sequences used in this study.
