# Supplementary material for: NPF-Driven Gart Expression Fuels Gut Absorption and Modulates Feeding via a Negative Feedback Loop
Source: Insects. 2026 May 21;17(5):528. doi: 10.3390/insects17050528 (PMC13207844; doi:10.3390/insects17050528)
Supplement: Supplementary file 1 [file insects-17-00528-s001.zip › insects-4302482-supplementary.pdf]

Table S1 Primer sequences used in this study

Primer	Sequences (5'-3')
Actin-F	CGGTGCAGTCACAAACGG
Actin-R	GTTGTTCTGGTTGCCATTATTCA
Gart-F	CACCGATGGCAAAAGTG
Gart-R	CATGAGTCCCGCATAGAG
NPF-F	CCGCTGTTCGTCTACAAGGA
NPF-R	TCCTGGTTGCCTGTGTGG
